# Underwater localization system for marine seismic airgun arrays validated through robotics

**DOI:** 10.1007/s41315-025-00429-3

**Published:** 2025-03-06

**Authors:** Ulises Tronco Jurado, Peter Wilson, Philippe Blondel, Andrew Bartin, Greg Walker-Doyle

**Affiliations:** 1 Department of Electronic and Electrical Engineering, University of Bath, Claverton Down, Bath, BA2 7AY UK; 2 Department of Physics, University of Bath, Claverton Down, Bath, BA2 7AY UK; 3 Seamap UK, Ltd, Unit 34, The Maltings Charlton Estate, Shepton Mallet, Somerset, BA4 5QE UK

**Keywords:** Marine seismic surveys, Airguns, Inertial/acoustic sensor system, Localization system, Sensor fusion

## Abstract

Marine seismic surveys rely on the precise localization of seismic airguns to ensure high-quality data acquisition. The current state-of-the-art systems for airgun localization, such as Ultra-Short Baseline (USBL), Long Baseline (LBL), and GPS-aided and hybrid systems, provide reliable positioning but are often costly, operationally complex or limited to surface tracking. To address these limitations, this research presents a novel, cost-effective, and robust underwater localization system designed explicitly for real-time trajectory tracking of seismic airguns. The proposed system integrates a low-cost Inertial Motion Unit (IMU), an open-source, modular, and low-power acoustic modem, and a depth sensor, employing an Extended Kalman Filter (EKF) using Robot Operating System (ROS) for the development of the sensor fusion and localization algorithm. The system achieves a position accuracy within 0.3–2 m, meeting the tolerances required for seismic surveys. Compared to USBL + INS and GPS-aided systems, the proposed system provides comparable precision while significantly reducing deployment complexity and operational costs. Unlike LBL systems, it does not rely on pre-installed seabed transponders, enhancing adaptability to different operational environments. Furthermore, its capacity to operate underwater without surface GPS dependency overcomes the limitations of existing systems in deep-sea or complex acoustic environments. The system is expected to enhance marine seismic data quality by enabling real-time positioning tracking, reduce marine seismic exploration times, and mitigate potential environmental impacts on marine ecosystems. This innovation bridges the gap between precision, affordability, and environmental sustainability in marine seismic exploration, making it a promising alternative for integrating trajectory position estimates of airguns into seismic survey workflows, helping to enhance the efficiency and effectiveness of marine seismic surveys worldwide.

**Supplementary Information:**

The online version contains supplementary material available at 10.1007/s41315-025-00429-3.

## Introduction

Marine seismic activity is widely employed in seafloor and subsurface mapping campaigns for commercial and scientific purposes. In the oil and gas sector, it is primarily used for hydrocarbon exploration in marine geosciences to detect and mitigate geohazards. In contrast, in related studies, the main focus has been determining the crust and upper mantle structures (Lurton 2002; Dondurur 2018; Khodabandeloo and Landrø 2018). In ocean exploration, airguns are the most commonly adopted seismic source due to several advantageous characteristics such as efficiency, environmental friendliness, high repeatability, and versatility in data collection. They provide improved signal quality, are cost-effective, and have a reduced impact on marine life compared to explosive sources. Moreover, airguns are suitable for urbanized areas and can be used with modern seismic imaging techniques (Staples and Hobbs 2001). These devices release high-pressure air (~ 13 MPa) from a chamber, generating low-frequency acoustic signals (up to 300 Hz) that propagate deeply into the seabed (Caldwell and Dragoset 2000; Krail 2010).

Despite their advantages, airguns face challenges such as the well-known bubble effect caused by the rapid release of compressed air, leading to acoustic disturbances (Lurton 2002; Dondurur 2018). Additionally, the harsh conditions of the oceanic environment and the continuous deployment of airguns can cause failures and, subsequently, unexpected downtime or lower-quality data. Real-time tracking of airgun performance and position is critical to ensure high-quality seismic data. Accurately determining both absolute and relative positions of airguns in an array is crucial for marine seismic surveys, as these positions influence the fidelity and consistency of seismic data acquisition. Properly aligned and synchronized airgun arrays ensure the generation of identical acoustic pulses, reducing noise and enhancing subsurface imaging quality. However, underwater localization is inherently challenging due to the inability to rely on a global navigation satellite system (GNSS), a global positioning system (GPS) or radio communications caused by the high attenuation of electromagnetic waves in water.

Over the last decade, researchers have developed and studied different underwater localization techniques and systems focused on autonomous underwater vehicles (AUVs) (González-García et al. 2020). These underwater localization methods can be divided into traditional and nontraditional methods using different approaches. Conventional methods, such as inertial navigation systems (INS) (iXblue. “Hydrins and Phins INS” 2025) and dead-reckoning (DR), estimate positions using velocity, acceleration, and water speed data. However, these methods suffer from accuracy drift over time due to environmental factors and sensor limitations (Leonard et al. 1998). Nontraditional methods leverage geophysical, acoustic, or optical technologies utilized to compensate for the accuracy drift of traditional methods. Geophysical localization uses sensor data compared with geophysical maps (Rice et al. 2004). Acoustic localization methods, including Long Baseline (LBL) (Zhang et al. 2016, 2019; Sonardyne 2025), Short Baseline (SBL) (Zhang et al. 2019; Yuyi et al. 2009), Ultra-Short Baseline (USBL) (Luo et al. 2020; Reis et al. 2016; Maritime 2025), inverted USBL (iUSBL) (Rypkema et al. 2017; Jakuba et al. 2015), and passive inverted USBL (piUSBL) (Rypkema and Schmidt 2019), utilize acoustic transponders for localization. While accurate, these systems are expensive and complex and often require specialized equipment for calibration and alignment. Their operational distances vary from 50 to 2000 m, 20 to 50 m, and less than 30 cm for the LBL, SBL, and USBL, respectively (Tan et al. 2011). Acoustic systems such as USBL are commonly used for airgun localization but are limited by surface-based dependencies and signal degradation in deep or acoustically noisy environments. On the other hand, LBL systems provide high precision with sub-meter accuracy but require complex pre-deployment setup and maintenance of seabed transponders, making them less flexible for dynamic survey conditions.

Optical technologies confront different challenges due to limited underwater illumination but continue to advance through optical sensor arrays (Eren et al. 2017) or cameras (Zhong et al. 2019). However, they remain less feasible for widespread use in airgun localization due to poor light transmission and visibility in deep-sea environments. Recent developments have introduced hybrid localization systems integrating multiple sensors (Sonardyne. 2025; Maritime 2025), such as Doppler velocity loggers (DVLs), depth sensors, and Inertial Measurement Units (IMUs) with acoustic sensors, to improve reliability and accuracy (Nicosevici et al. 2004; Xing et al. 2021; Yan et al. 2018). These systems aim to compensate for the limitations of individual technologies but often result in increased system complexity and cost. Current seismic acquisition systems employ sophisticated navigation software to estimate positions for vessels, streamers, airgun arrays, tail buoys, paravanes, and so on (Dondurur 2018). The real-time positioning of airguns is generally estimated using GPS antennas or hydrophone data inversion, achieving an accuracy of 0.2–3 m (Ni et al. 2012). However, these systems are surface-restricted and cannot provide consistent underwater tracking. GPS mounted on airgun subarrays can only estimate positions at the surface, leaving underwater trajectories dependent on indirect calculations, which may introduce errors under challenging environmental conditions.

The proposed airgun localization system in this research paper addresses these limitations by integrating a low-cost, open-source, modular, and energy-efficient acoustic modem network, an IMU, and a depth sensor with an Extended Kalman Filter (EKF) (Radojevic and Superior 2011; Moore and Stouch 2016) for data fusion. Unlike traditional LBL or USBL configurations, the system achieves comparable precision (within the 0.2–3 m range) without requiring extensive infrastructure, making it more adaptable and cost-effective for dynamic marine survey conditions. The system’s use of EKF-based sensor fusion minimizes drift errors commonly associated with INS-based localization by continuously correcting the estimated trajectory with acoustic positioning and depth sensor data. This hybrid approach is intended to ensure high accuracy in both absolute and relative positioning, aligning with operational tolerances for marine seismic data acquisition with a maximum allowable positioning error for airgun strings of 2 m (Dondurur 2018) Additionally, it provides real-time trajectory tracking, improving operational consistency. While hybrid systems in current use offer accuracy, their reliance on costly components and complex setups often limits scalability and ease of deployment. In contrast, the proposed system’s low-cost design and modularity intend to ensure ease of integration into existing marine seismic acquisition workflows.

This research demonstrates the airgun localization system performance and effectiveness through controlled experiments, leveraging a universal robot (UR3) under air conditions and an XY linear robot in underwater conditions to simulate airgun dynamic motion (Khodabandeloo and Landrø 2018; Caldwell and Dragoset 2000; Krail 2010). The results indicate that the proposed system could help enhance marine seismic data quality by reducing positional uncertainty. It can also help minimize marine survey durations and increase operational reliability, offering a practical alternative to state-of-the-art airgun localization systems. The following sections detail the airgun positioning system design in Sect. 2, the EKF localization algorithm setup in Sect. 3, and the system testing, characterization, and performance analysis in Sect. 4. Conclusions and future directions are discussed in Sect. 5.

## Airgun positioning system description and design

### Inertial motion unit (IMU)

**Fig. 1:**
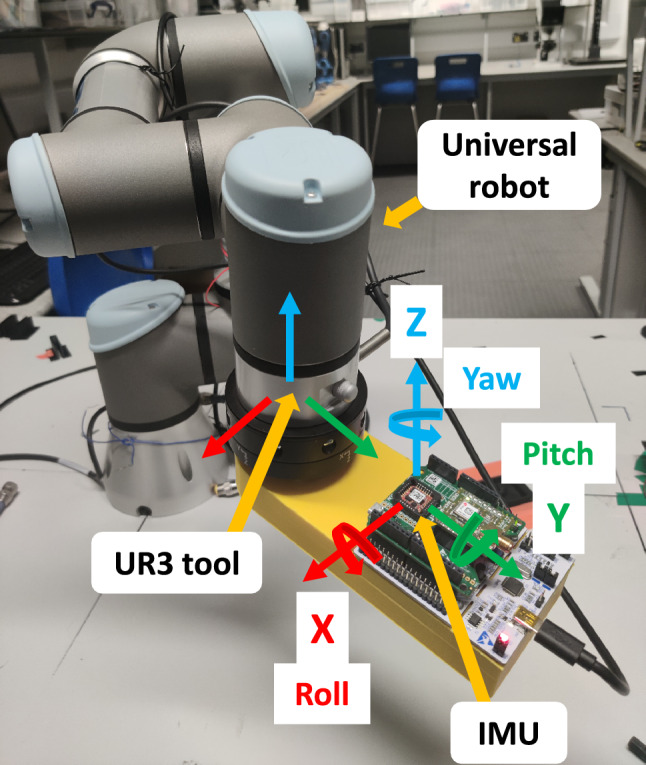
The precision and accuracy of the IMU orientation against the UR3 tool were characterized through different simulations and velocities

Inertial measurement technology has become an attractive methodology due to the reduced production cost and performance enhancement of the sensing components. Nonetheless, inertial measurement technology has limitations when it is employed to track sensor position due to a growing position drift. The pose and velocity of the airgun localization system were selected using an Xsens MTi-7 after the performance analysis against other low-cost IMUs through a simulated controlled motion using the universal robot depicted in Fig. 1.

The RMS values for roll, pitch, and yaw orientation angles determined through different motion simulations and velocities compared against the point tool of the UR3 showed the most precise values of 1.25 ± 0.93, 3.67 ± 0.76, and 0.59 ± 0.79, respectively, with accuracies of 98.32%, 92.79%, and 99.47%, respectively, as depicted in Table 1.

**Table 1:** Average roll, pitch, and yaw RMS values of different IMUs characterized using the universal robot UR3 tool as a baseline through various simulations and velocities

IMU	RMS values		
	Roll °	Pitch °	Yaw °
Xsens Mti-7	73.12 ± 023	47.33 ± 0.50	111.54 ± 0.48
BNO055	66.42 ± 0.41	44.61 ± 0.46	119.23 ± 0.73
ICM20948	73.04 ± 0.30	35.77 ± 0.36	132.19 ± 0.31
MPU9250	71.54 ± 0.68	37.67 ± 0.56	106.15 ± 0.30
UR3 (Baseline)	74.36	51	112.33

These results demonstrated that the Xsens Mti-7 is a reliable inertial sensor system that combines cutting-edge technology, high precision, and flexibility for integration into an airgun localization system because of its compact, low-cost, lightweight inertial navigation module offering precise motion tracking with its magnetometer, accelerometer, gyroscope, and external GNSS integration. It features advanced sensor fusion algorithms for accurate orientation and positioning, low drift, high update rates, and temperature calibration. With flexible interfaces such as I2C, UART, SPI, and CAN, it can be adapted to diverse use cases, making it a versatile and reliable solution for demanding localization and navigation tasks (Crabolu et al. 2018). However, when using IMU sensors in an underwater environment, several considerations, such as magnetic interference, water pressure and temperature, laboratory in-field calibration and out-field online calibration (Poddar et al. 2017; Wu et al. 2024), mounting and orientation, sensor selection, and compensation for errors must be considered to ensure accurate measurements and optimal performance. The selected Xsens Mti-7 sensor was out-field online calibrated (Poddar et al. 2017; Wu et al. 2024) using the magnetic field mapper and MT SDK program from Xsens. The sensor initialization process was verified and monitored before being tested and integrated with the airgun localization system using the UR3 robot and the XY linear robot tool in underwater conditions to ensure its ideal performance, as described in Sect. 4.

### Acoustic modem

**Fig. 2:**
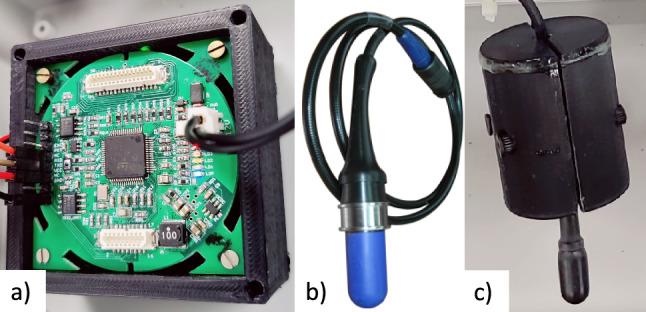
**a** Ahoi modem, **b** Nearfield Hydrophone from Seamap Ltd., and **c** AS-1 hydrophone from Aquarian

Acoustic underwater communication has recently been widely used among research groups and companies. However, there is a high cost of commercial acoustic modems, e.g., Sonardyne AvTrak 6 Nano (Sonardyne 2025) and Evologics S2C M HS (GmbH 2025), from £9 K to £12 K per unit. For that reason, a microcontroller (µC)-based, low-cost (£600), low-power, open-source, and small Ahoi Modem (Renner et al. 2020; Busse and Renner 2022) was used for integration into the airgun localization system to measure distance data and calculate position. The modem consists of three stacked boards: the mainboard, receiver, and transmitter, which are 50 mm × 50 mm × 22 mm, and an external hydrophone connected to the transmitter board, as depicted in Fig. 2a. The modem is compatible and can be adjusted to work with various hydrophones and frequency bands (typical 24 to 60 kHz) with data rates ranging from 250 to 4500 bits per second and a range of communication up to 300 m.

Communication with the Ahoi modem is packed; however, it supports distance measurement employing two-way time-of-flight ranging (TWR) (Renner 2017) between acoustic modems. A distance measurement is activated by sending a data packet (ahoi packet format (Renner et al. 2020)) from one modem to another modem/modems or as a broadcast. The second modem replies to an acknowledgment (ACK), and then the first modem receives the ACK, which calculates the acoustic wave propagation time. A packet handler processes this packet payload for distance conversion. The transducers used with the acoustic modems acting as both the sender and receiver were the Near Field Hydrophones from Seamap Ltd. (Seamap Ltd. 2022) and the AS-1 hydrophones from Aquarian (Scientific. 2022) (Fig. 2b-c), both of which have good omnidirectionality. The ranging accuracy of the acoustic modems was tested using 100 ranging requests per direction for each pair of anchors before the start of the test using the XY linear robot tool in underwater conditions. This analysis is required for the validity of mobile localization because the latter is only feasible if ranging in the static case is reliable and sufficiently precise (Renner et al. 2020).

### Sensor fusion prototype and experimental setup

**Fig. 3:**
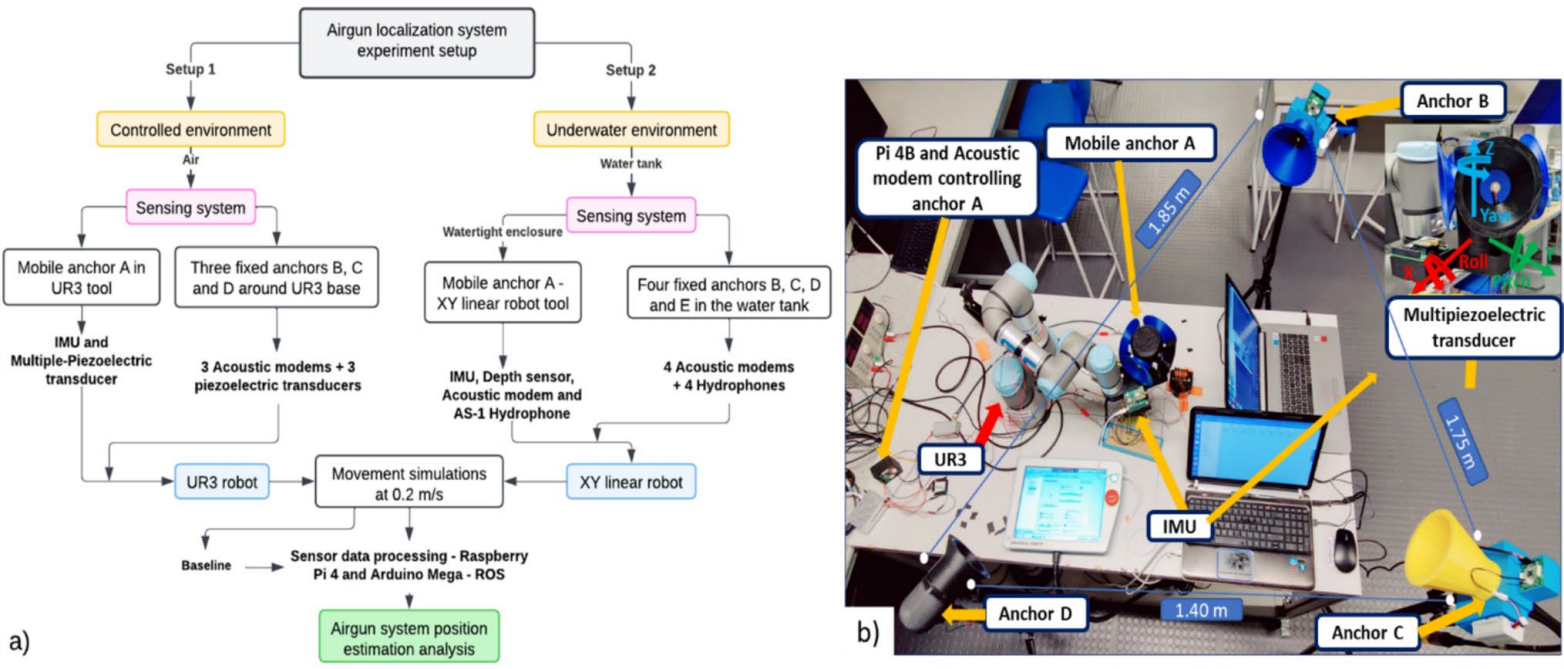
**a** Flow chart of the airgun localization system experimental process setup. **b** Experimental setup one evaluates the airgun localization system employing a UR3 robot that simulates different motion movements in a controlled environment

To evaluate the sensor fusion data performance of the airgun localization system comprising the IMU and acoustic modems, the system was assessed through two experimental setups illustrated in Fig. 3a. First, a mobile anchor comprising the IMU and a multiple-piezoelectric transducer (Murata, SMD Diaphragm) controlled by an acoustic modem was attached to the tool at UR3, as illustrated in Fig. 3b. Then, three fixed anchors were located at different points around the base of UR3 (Fig. 3b). The airgun system performance was evaluated using the movement of the UR3 tool as the baseline through different movement simulations in a controlled environment (Fig. 3b) with an average speed of 0.2 m/s.

**Fig. 4:**
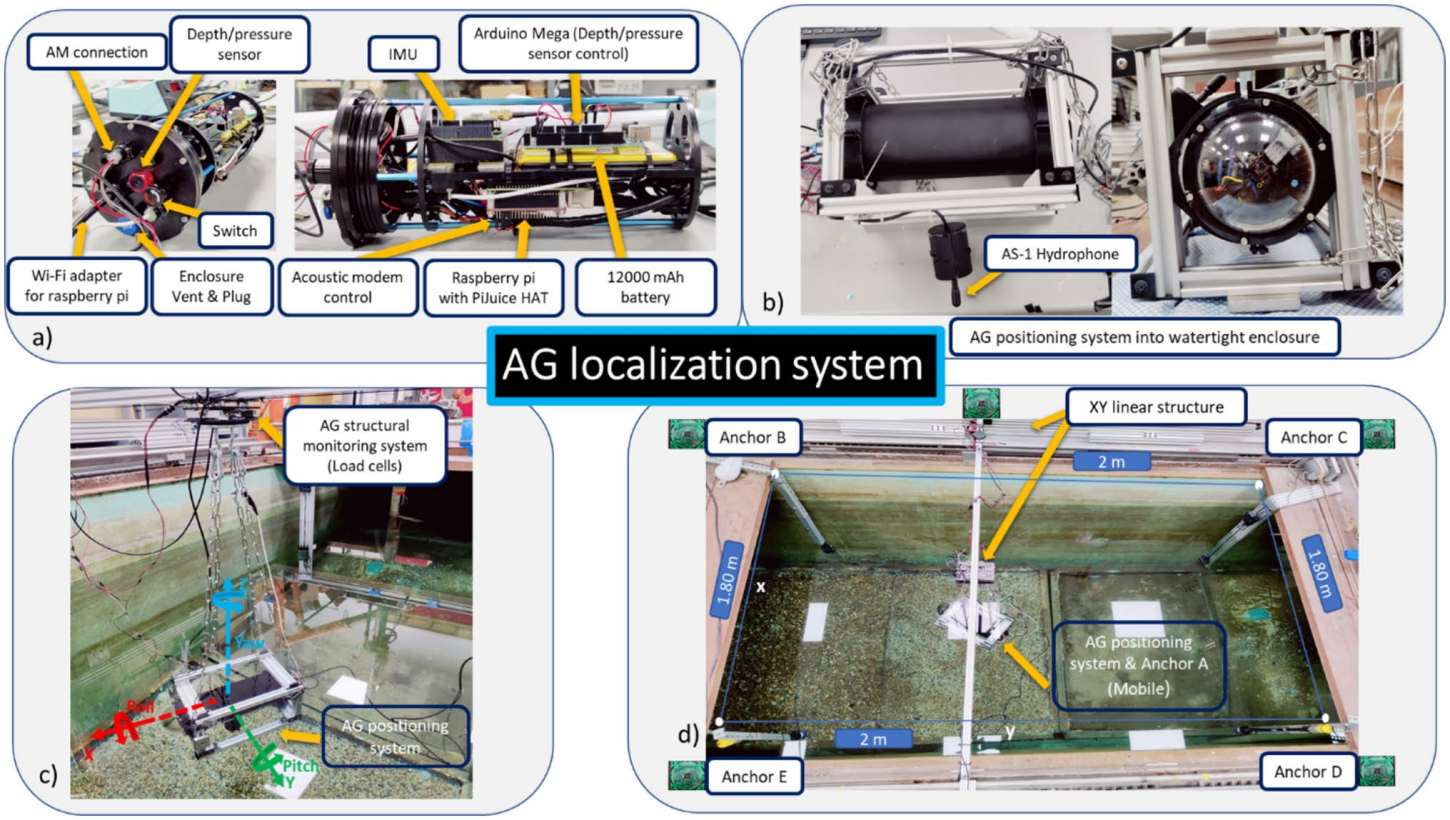
Experiment setup for evaluating the airgun localization system employing an XY linear robot simulating different motion movements under underwater conditions

Second, to test the system’s performance under underwater conditions, a water tank with a length = 2 m, width = 2 m, and depth = 2 m was utilized (Fig. 4d). A four-inch aluminum watertight enclosure was used to protect all the sensor fusion circuitry, as depicted in Fig. 4a-b. The enclosure acts as the mobile anchor, comprising the IMU, an acoustic modem connected to an AS-1 hydrophone, a Bar02 ultrahigh-resolution depth/pressure sensor, and an Arduino Mega to process the depth sensor data. Raspberry Pi 4 8 GB connected to a PiJuice HAT, a portable power platform powered by a 12,000 mAh battery, was used for all the sensor data processing, as illustrated in Fig. 4 a-b. Furthermore, remote communication with the Raspberry Pi was performed using an Archer T2U Wi-Fi adapter to ensure stable communication with the airgun localization system. Additionally, four acoustic modems connected to AS-1 and nearfield hydrophones were fixed through different positions in the water tank at a depth of 1.2 m for the packet exchange and positioning calculations once they communicated with the mobile anchor (Fig. 4d). The airgun system performance under underwater conditions was evaluated using the movement of the XY linear robot tool, which was utilized as the baseline tool through different simulations of movement with an average speed of 0.2 m/s (Fig. 4d).

## Configuration of the EKF localization algorithm

The sensor fusion data were processed through Robot Operating System (ROS) (Robot Operating System 2022), in which a ROS node was implemented for each sensor that publishes each sensor dataset using a ROS topic.

The position of the airgun system was estimated through different simulations of movement under air and underwater controlled conditions by implementing the robot localization ROS package. This approach provides nonlinear state estimation and can merge information from various kinds of sensors using the well-known extended Kalman filter (Radojevic and Superior 2011; Moore and Stouch 2016) to determine the state of a robot moving in a 3D environment in this case, simulating the movement of an airgun deployed underwater. Such a method can be defined as a nonlinear dynamic system as follows (Moore and Stouch 2016):

1 $$x_{k} = f\left( {x_{k - 1} } \right) + w_{k - 1}$$

where *x*_*k*_ is the 3D state of the robot at time *k*, *f* is a nonlinear state transition function, and *w*_*k-1*_ is the process noise with covariance *Q* that is expected to be normally distributed. *x* is a twelve-dimensional state vector that includes the robot’s 3D pose, 3D orientation, and respective velocities (Jakuba et al. 2015). In addition, measurements are related to the state by:

2 $$z_{k} = h\left( {x_{k} } \right) + v_{k}$$

where the measurement at time *k* is *z*_*k*_, which is a nonlinear sensor model that maps the state into the measurement space, is *h*, and *v*_*k*_ is the normally distributed measurement noise with covariance *R* (Leonard et al. 1998). In addition, the first phase of the algorithm is the implementation of a prediction step projecting the estimation of the current robot state, followed by an update step, and then a series of calculations are performed to promote filter stability by ensuring that the covariance remains positive semi-definite, as described in detail in Moore and Stouch (2016). The EKF prediction and update equations are the following:

3 $$\hat{x}_{k}^{ - } = f\left( {\hat{x}_{k - 1} } \right)$$

4 $$P_{k}^{ - } = F \, \times P_{(k - 1)} \, \times F^{T} + Q$$

where ${\widehat{x}}_{k}^{-}$ is the predicted state, ${P}_{k}^{-}$ the predicted state covariance and $F= {\left.\frac{\partial f}{\partial x}\right|}_{{\widehat{x}}_{k-1}}$ is the Jacobian of the state transition model.

5 $$K_{k} = P_{k}^{ - } \times H^{T} \left( {H \times P_{k}^{ - } \times H^{T} + R} \right)^{ - 1}$$

6 $$\hat{x}_{k} = \hat{x}_{k}^{ - } + K_{k} (z_{k} - H \, \times \hat{x}_{k}^{ - } )$$

7 $$P_{k} = (I - K_{k} \times H)P_{k}^{ - }$$

where ${K}_{k}$ is the Kalman gain, ${z}_{k}$ is the measurement vector, ${\widehat{x}}_{k}$ is the updated state estimate, and ${P}_{k}$ the updated state covariance. The standard EKF development stipulates that the observation matrix *H* should be a Jacobian matrix of the observation model function *h*. In essence, *H* is simply the identity matrix that supports a broad array of sensor measurements and variables that can be fused in the final state estimate using the robot localization package (Moore and Stouch 2016).

**Fig. 5:**
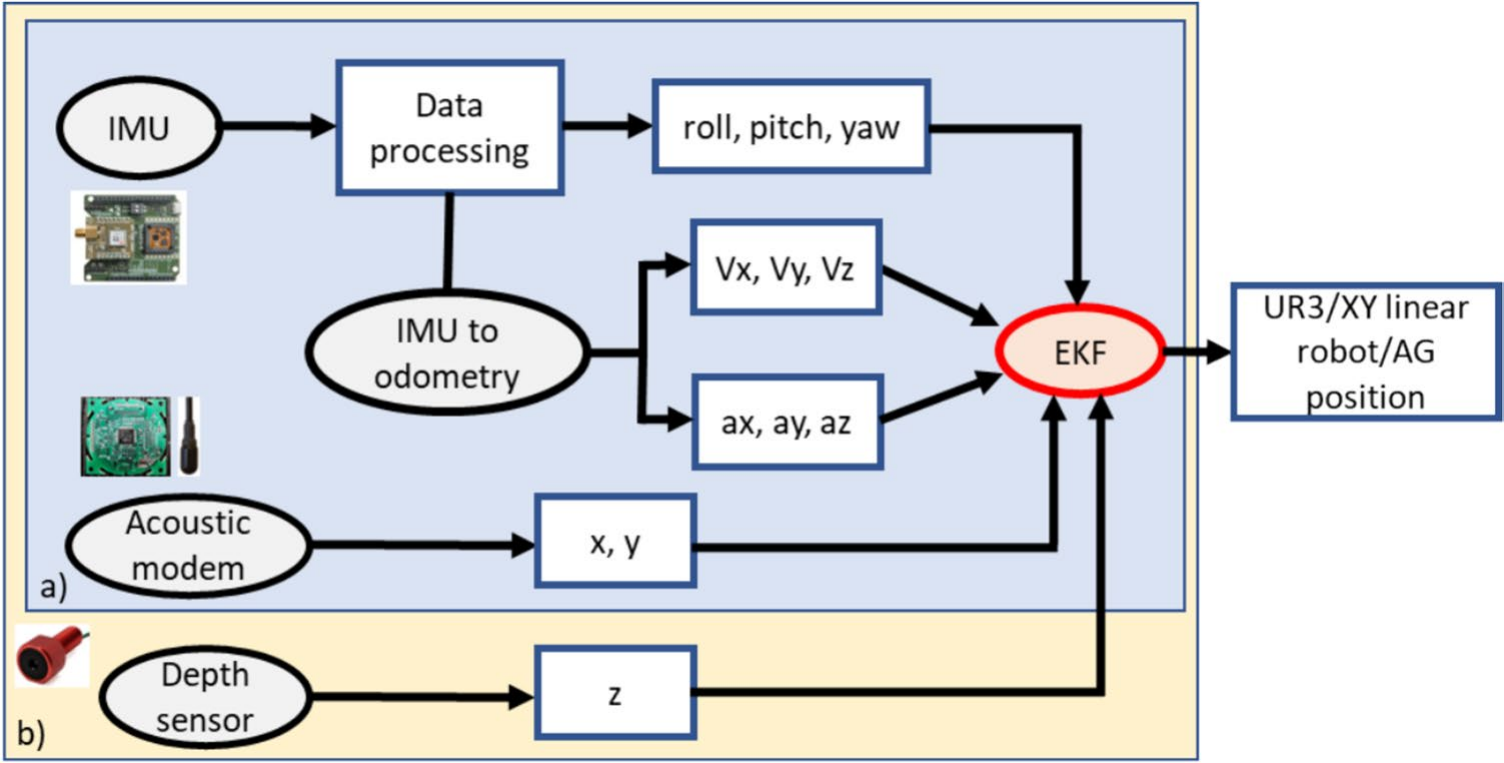
**a** Schematic diagram of the airgun localization system tested in UR3 in a controlled environment and **b** in XY linear motors under underwater conditions

The EKF filter is used to estimate the position of the tool of the UR3 robot in a controlled environment and to estimate the XY linear motor movement in underwater conditions over time through different simulations of movement. The state space (*x*) comprises the position *x*, *y*, *z*, orientation roll, pitch, yaw, linear velocities *d*x/dt, *d*y/dt, *d*z/dt, and angular accelerations roll/dt, pitch/dt, and yaw/dt. For each sensor, the variable configuration of the corresponding measurement data fused in the EKF through *H* was specified for the final estate estimation of the UR3 robot or XY linear robot, as depicted in Fig. 5 and Table 2 (See Appendix 1 document).

**Table 2:** Sensor configurations for the airgun localization system

Sensor	Configuration (0 = false, 1 = true)											
	x	y	z	roll	pitch	yaw	dx/dt	dy/dt	dz/dt	roll/dt	pitch/dt	yaw/dt
IMU	0	0	0	1	1	1	0	0	0	1	1	1
Acoustic modem	1	1	0	0	0	0	0	0	0	0	0	0
Odometry	0	0	0	0	0	0	1	1	1	0	0	0
Depth	0	0	1	0	0	0	0	0	0	0	0	0

The IMU measures orientation (roll, pitch, and yaw) and linear velocities, and the acoustic modem measures the position in x and y through a multilateration algorithm based on at least three anchors (Sonardyne. 2025; Tan et al. 2011; Eren et al. 2017; Zhong et al. 2019; Nicosevici et al. 2004; Xing et al. 2021; Yan et al. 2018; Ni et al. 2012; Radojevic and Superior 2011; Moore and Stouch 2016; Crabolu et al. 2018; GmbH 2025; Renner et al. 2020; Busse and Renner 2022) of several ranging measurements between a mobile anchor and fixed anchors with known positions (Figs. 3 and 4d). A single packet was used to collect distance from all fixed anchors. First, the mobile acoustic modem sends an empty-ranging request as a broadcast. Second, once the anchors receive the request, the anchors reply with a consecutive ranging response. Anchor A replied first, followed by anchors B, C, and D with a single, double, and triple delay (750 ms) (Renner et al. 2020; Busse and Renner 2022), respectively, to avoid interference between anchor-ranging responses. Every run lasted approximately 6 min; 102 ranging requests were sent with an interval of 3.5 s using the acoustic modem. Odometry is utilized in localization systems and is usually fundamental for tracking target device poses in a 3D unknown environment. The system’s odometry, such as linear velocities and angular accelerations, was estimated using IMU data (Czapiewska et al. 2020) (Fig. 5). Consequently, rough estimates of the UR3, XY linear robot, or airgun poses can be calculated. Additionally, the EKF employs a covariance matrix from each input sensor to estimate the uncertainty of each sensor data point, producing a covariance matrix for the localization of the system being tracked (See Appendix 1 document).

## Testing, characterization and analysis

**Fig. 6:**
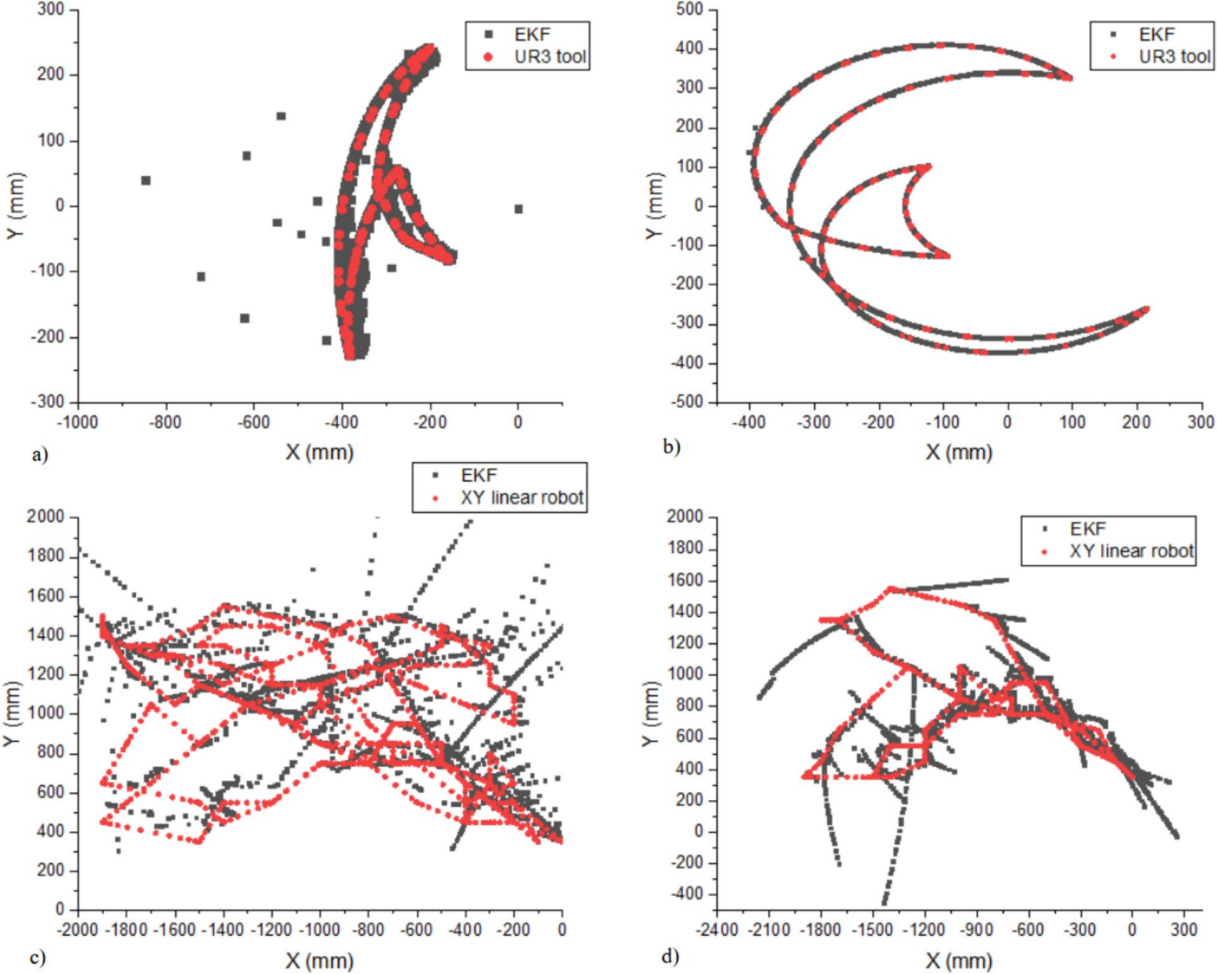
**a**–**b** Comparison of the trajectory position of the UR3 tool (baseline) in red and the estimated trajectory position in dark gray based on the airgun localization system tested in a controlled environment through different simulations of movement. **c**–**d** Comparison of the trajectory position of the XY linear robot tool (baseline) in red and the estimated trajectory position in dark gray based on the airgun localization system tested in underwater conditions (see Appendix 1 document illustrating the localization error over time and Appendix Video 1)

The performance analysis of the airgun localization algorithm tested in two different controlled environments demonstrates varying levels of performance analyzed across different precision thresholds as illustrated in Table 3 (See Appendix 1 describing the localization error over time). First, in air conditions, tracking the trajectory position of the UR3 robot tool while performing different motion simulations is illustrated in Fig. 6a–b. The difference between the baseline and the estimated trajectory position shows that less than half of the measurements achieve sub-100 mm accuracy and show excellent reliability within the 500 mm threshold in most cases (Table 3). Furthermore, the system tested under the previously mentioned conditions showed an overall average precision of 90.29 ± 126.74 mm and 236.01 ± 191.42 mm when analyzing the whole estimated trajectory data in the X and Y axes, respectively, compared with the baseline trajectory of the UR3 tool, as depicted in Fig. 6a–b and Table 3. The precision analysis in the X axis shows good average precision with moderate variability, lower precision, and higher variability in the Y axis. Overall, it shows better precision in the X than Y axis. As a result, the system’s accuracy within a threshold of 100 mm to 500 mm was between 42.40% and 99.90%, respectively, for X and between 31.94% and 85.80% for Y. The error percentage analysis of X (31.94%) and Y (36.94%) show a relatively balanced error distribution. Overall, the system tested in air conditions shows a total precision X–Y of 298.41 ± 150.29 mm (Table 3). Such results establish a baseline performance metric, showing the system’s capabilities under controlled conditions.

**Table 3:** Precision, accuracy, and error of the trajectory position estimation of the airgun localization system tested in different environments through simulated motions and analyzed within different thresholds (See Appendix 1 document illustrating the localization error over time)

Airgun localization algorithm performance								
Baseline	Axis		Precision (mm)	Error %	Accuracy % (within 100 mm)	Accuracy % (within 500 mm)	Accuracy % (within 1000 mm)	Accuracy % (within 2000 mm)
UR3 tool	Y		90.29 ± 126.74	31.94	42.40	99.90	100.00	100.00
	X		236.01 ± 191.42	36.10	31.30	85.80	100.00	100.00
	Total X–Y		298.41 ± 150.29	34.74	12.80	83.65	100.00	100.00
XY linear robot tool	X		130.71 ± 379.81	38.82	53.15	70.35	88.30	100.00
	Y		27.62 ± 370.34	41.54	55.60	73.50	95.95	99.30
	Total X–Y		475.84 ± 269.93	34.74	50.65	60.2	79.25	98.85

Second, the estimation of the trajectory position tested in underwater conditions of the localization system was compared with the trajectory obtained by the XY linear robot (Fig. 6c–d), which was used as the baseline and analyzed within different thresholds (Table 3). The system achieves notable high-precision performance, with accuracy rates of 53.15% and 55.60% for the X and Y axes, respectively, at the 100 mm threshold, interestingly achieving better high precision underwater than the test in air conditions. This indicates good capability for delicate positioning tasks. The system maintains reliable performance at ranges between 500–1000 mm, with accuracy rates above 70% for both axes. The system excels at the 2000 mm threshold, achieving nearly perfect accuracy (100% for the X-axis and 99.30% for the Y-axis). The average precision measurements on the X and Y axes of 130.71 ± 379.81 mm and 27.62 ± 370.34 mm, respectively, indicate that while the system can achieve good average precision, especially in the Y-axis, there is significant measurement variability. The error percentages of 38.82% for the X-axis and 41.54% for the Y-axis suggest consistent performance across both axes, also reflected in the system’s total precision X–Y (475.84 ± 269.93 mm). However, there is room for improvement in overall precision and accuracy (Fig. 6c–d and Table 3).

The air and underwater testing comparison reveals the airgun localization system’s robust design and adaptability. While underwater conditions introduce more significant variability in measurements, as seen in the standard deviations (Table 3), the relatively small performance drop between air and underwater testing of approximately 25–30% at medium ranges (between 500–1000 mm) indicates strong system resilience. The high variability in the trajectory position calculation is related to two factors. First, the position and alignment of the fixed hydrophones (Renner et al. 2020) in the water tank were determined. These were placed in proximity to walls made of concrete, which causes some reflections when the acoustic modems are in ranging communication, exchanging packets. Such an effect can be seen in Fig. 6c–d, which shows a drift error in the trajectory position estimations on the X-axis and Y-axis near the concrete walls of the tank, reducing the quality of the estimation data. To minimize the reflections during underwater communication using the hydrophones and acoustic modems, some potential strategies that can be implemented as future work are an optimal placement to reduce interactions with reflective surfaces and testing in a bigger water tank (Renner 2017; Czapiewska et al. 2020), adaptive filtering for real-time cancelation of reflections (Czapiewska et al. 2020), beamforming to focus on direct signals (Quartly and Pace 1991), and frequency tuning to match environmental conditions (9.^1^ Acoustic communication principles and limitations 2024). Additionally, error correction protocols (Gupta and Williams 2013) and multipath-resilient modulation like orthogonal frequency division multiplexing (OFDM) further enhances data reliability (Zhang et al. 2021). Using sound-absorbing materials on surfaces can also reduce reflections (Bernard et al. 2022). These approaches could help significantly improve communication reliability in reflective underwater environments. Second, the IMU-based odometry approach employed through the localization algorithm using the low dimensional inertial data from the IMU can cause a critical drift error over time (Silva do Monte Lima et al. 2019). This could be part of the cause of the drift error shown in Fig. 6c–d in the X and Y axes of the trajectory position estimation underwater conditions. To reduce such drift errors, different IMUs were tested, as described in Sect. 1. The Z-axis data under underwater conditions were controlled with a depth sensor, which provided constant data indicating that the localization system was at ~ 45 cm depth during the experiment. Practical strategies that can be implemented in future work to mitigate the time-critical drift error in the IMU-based odometry in this airgun localization system that uses acoustic multilateration and EKF-based fusion are optimize anchor placement (Renner 2017; Czapiewska et al. 2020), model acoustic range and IMU biases (Skog and Handel 2009), and employ tightly coupled fusion strategies (Thrun et al. 2005). Periodic corrections with zero-velocity updates (ZUPTs) (Yuan et al. 2024), integrating DVLs (Liu et al. 2020), and terrain reference navigation (TRN) (Eustice et al. 2005) can provide additional stabilization. Lastly, machine-learning approaches (Cohen and Klein 2024) offer innovative solutions for real-time drift prediction and correction.

**Fig. 7:**
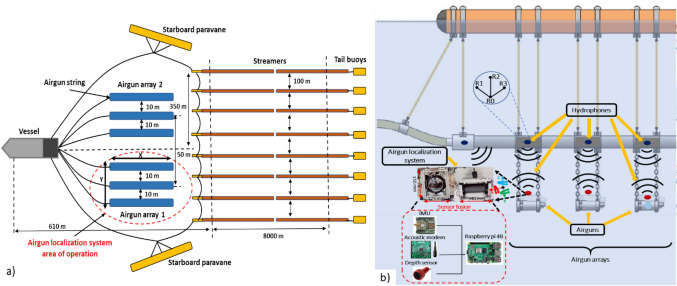
** a** Schematic diagram of a 3D seismic tow set up with two airgun arrays, eight streamers, and tail buoys, highlighting the airgun localization system’s operation area. **b** A lateral view of an airgun string and nearfield hydrophones illustrates the proposed area where the airgun localization system can be installed for future deployment and testing in field conditions/marine environments during marine seismic surveys

As a result, the maintenance of high accuracy rates (> 88% at a 1-m threshold) in both conditions, combined with better-than-expected high-precision performance in underwater conditions, demonstrates the system’s suitability for the integration of an airgun localization system under real conditions deployed in the ocean, showing potential to achieve high accuracy at sub-meter ranges (Table 3). The size and area of the airgun/airgun arrays deployed underwater to acquire marine seismic data (seismic surveys), where the proposed airgun localization system is intended to be installed, is illustrated in Fig. 7a-b. The variations in the precision of the X (130.71 mm ± 379.81 mm) and Y (27.62 mm ± 370.34 mm) trajectories could be highly accurate due to the large area of operation of the airgun deployed underwater (approximately 20 m) (Fig. 7a). The distance between the airgun strings is ~ 10 m, and between airgun arrays is ~ 50 m. This means that the airgun localization system proposed and tested in this research study has the potential to track the airgun trajectory position in real-time. Additionally, the precision of the proposed airgun localization system is within the precision range of 0.2–3 m, which is reached by the existing localization system used to detect airguns mentioned in Sect. 1 and illustrated in detail in Table 4. Additionally, within the maximum allowable positioning error of 2 m for airgun strings, this range corresponds to the accepted tolerances for seismic data acquisition (Dondurur 2018).

**Table 4:** Airgun localization system comparison against existing underwater localization systems

System	State-of-the-art solutions	Proposed system
	Precision	Precision
USBL	1–2 m depending on water depth and acoustic conditions (Sonardyne. 2025; Luo et al. 2020; Reis et al. 2016; Maritime 2025)	Comparable precision (0.3–2 m) under similar conditions
LBL	Sub-meter precision in well-calibrated systems (Zhang et al. 2016, 2019; Sonardyne. 2025)	It is slightly less precise but still within acceptable tolerances for seismic surveys
INS	High temporal accuracy but prone to drift over time without correction (iXblue. "Hydrins and Phins INS." 2025)	Combines INS with acoustic data and EKF to reduce drift, achieving stable performance
GPS-aided	0.2–3 m limited to surface applications (Ni et al. 2012)	Matches or outperforms GPS-aided systems by working underwater
Hybrid systems	Sub-meter precision due to high-quality sensor integration (Sonardyne. 2025; Maritime 2025)	Achieves acceptable accuracy while using cost-efficient components

## Conclusion

During marine seismic surveys, high-quality data must be acquired. However, due to harsh oceanic conditions, the continuous deployment and operation of airguns in such marine surveys can cause failures reflected in low-quality data acquisition. Accurate real-time localization of airguns is essential to ensure consistent marine seismic source positioning, meeting the operational requirement for a maximum positioning error of 2 m to maintain data quality. Addressing these challenges, the developed and characterized airgun localization system offers a low-cost, open-source, modular, robust, and effective alternative solution to current state-of-the-art systems. The proposed system employs a sensor fusion inertial/acoustic approach processed through an Extended Kalman Filter. The achieved accuracy of 0.3–2 m (Ni et al. 2012) aligns with the precision of existing localization systems (Table 4) and within the maximum allowed positioning error of 2 m for airgun strings, corresponding to the accepted tolerances for seismic data acquisition (Dondurur 2018). These results are promising for the future integration of the system into airgun arrays (Fig. 7b), facilitating and potentially estimating the trajectory position of airguns in real-time during marine seismic surveys. As a result, inputting the trajectory position estimates of airguns into marine seismic data acquired during surveys is expected to enhance the quality of the data and potentially reduce the duration of marine seismic surveys in the field, which usually can take twelve months (Barkaszi and Kelly 2018). If survey time can be reduced, this not only optimizes operational costs but also minimizes the potential long-term impacts on marine life (Carroll et al. 2017), addressing environmental concerns associated with extended seismic surveys. Furthermore, the system’s lower cost and simplicity in deployment position it as an accessible alternative for widespread use in marine seismic surveys. However, future work and further testing should focus on deploying the proposed airgun localization system in real-world oceanic or river conditions to validate its robustness under dynamic environments and high-pressure scenarios generated by airgun shooting during marine exploration surveys. Additionally, incorporating visual odometry data and GNSS-based surface localization into the sensor fusion algorithm could enhance accuracy and mitigate drift errors commonly associated with INS-based localization. These advancements, coupled with previously mentioned strategies to mitigate acoustic data exchange reflections, position this system as a transformative solution in marine seismic exploration, ensuring higher efficiency, precision, and sustainability in future marine surveys.

## Supplementary Information

Below is the link to the electronic supplementary material.

Supplementary file1 (PDF 1070 KB)

Supplementary file2 (MP4 37170 KB)

## Data Availability

No datasets were generated or analysed during the current study.
