# Supplementary material for: Underwater localization system for marine seismic airgun arrays validated through robotics
Source: Int J Intell Robot Appl. 2025 Mar 6;9(3):1255–67. doi: 10.1007/s41315-025-00429-3 (PMC12507950; doi:10.1007/s41315-025-00429-3)
Supplement: Supplementary file 1 — Supplementary file1 (PDF 1070 KB) [file 41315_2025_429_MOESM1_ESM.pdf]

Supplementary Information

1 – Mathematical representation of the Extended Kalman Filter (EKF) used for the airgun localization system.

The system fuses data from an acoustic modem (positions *x* and *y* (m)*)*, an IMU (orientation *roll*, *pitch*, *yaw* (radians), linear velocities *x/dt*, *y/dt*, *z/dt* (m/s) and angular accelerations *roll/dt*, *pitch/dt* and *yaw/dt* (rad/s^2)) and a depth sensor (depth *z* (m)) to estimate the position of marine seismic airguns/airgun arrays.

The system's variables on the state vector *x* and measurement vector *z* are defined as:

$$x = z = \left[x\ y\ z\ roll\ pitch\ yaw\ \frac{x}{dt}\frac{y}{dt}\frac{z}{dt}\frac{roll}{dt}\frac{pitch}{dt}\frac{yaw}{dt}\right]^T$$

In the EKF, the state transitional model evolves as follows:

$$x_k = f(x_{k-1}) + w_{k-1} \tag{1}$$

where *xk* is the 3D state of the robot at time *k*, *f* is a nonlinear state transition function, and *wk-1* is the process noise with covariance *Q* that is expected to be normally distributed. *x* is a twelve-dimensional state vector that includes the robot's 3D pose, 3D orientation, and respective velocities [19]. In addition, measurements are related to the state by:

$$z_k = h(x_k) + v_k \tag{2}$$

where the measurement at time *k* is *zk*, which is a nonlinear sensor model that maps the state into the measurement space, is *h*, and *vk* is the normally distributed measurement noise with covariance *R* [9]. In addition, the first phase of the algorithm implements a prediction step, projecting the estimation of the current robot state, followed by an update step. Then, a series of calculations are performed to promote filter stability by ensuring that the covariance remains positive semi-definite, as described in detail in [29]. The EKF prediction and update equations are the following:

$$\hat{x}_k^- = f(\hat{x}_{k-1}) \tag{3}$$

$$P_k^- = F \times P_{k-1} \times F^T + Q \tag{4}$$

Where $\hat{x}_k^-$ is the predicted state, P_k^- the predicted state covariance and $F = \frac{\partial f}{\partial x}\Big|_{\hat{x}_{k-1}}$ is the Jacobian of the state transition model.

$$K_k = P_k^- \times H^T(H \times P_k^- \times H^T + R)^{-1} \tag{5}$$

$$\hat{x}_k = \hat{x}_k^- + K_k(z_k - H \times \hat{x}_k^-) \tag{6}$$

$$P_k = (I - K_k \times H)\, P_k^- \tag{7}$$

Where K_k is the Kalman gain, z_k is the measurement vector, $\hat{x}_k$ is the updated state estimate, and P_k the updated state covariance.

The standard EKF development stipulates that the observation matrix *H* should be a Jacobian matrix of the observation model function *h*. In essence, *H* is simply the identity matrix that supports a broad array of sensor measurements and variables that can be fused in the final state estimate using the robot localization package [29]. Since all measurements correspond directly to state variables, the observation matrix *H* for the airgun localization system is a 12 x 12 identity matrix as follows (Illustrated in the manuscript in Table 2. Sensor configurations for the airgun localization system for better visualization) :

$$H = I_{12} = \begin{bmatrix} 1&0&0&0&0&0&0&0&0&0&0&0\\ 0&1&0&0&0&0&0&0&0&0&0&0\\ 0&0&1&0&0&0&0&0&0&0&0&0\\ 0&0&0&1&0&0&0&0&0&0&0&0\\ 0&0&0&0&1&0&0&0&0&0&0&0\\ 0&0&0&0&0&1&0&0&0&0&0&0\\ 0&0&0&0&0&0&1&0&0&0&0&0\\ 0&0&0&0&0&0&0&1&0&0&0&0\\ 0&0&0&0&0&0&0&0&1&0&0&0\\ 0&0&0&0&0&0&0&0&0&1&0&0\\ 0&0&0&0&0&0&0&0&0&0&1&0\\ 0&0&0&0&0&0&0&0&0&0&0&1 \end{bmatrix}$$

The process noise covariance (*Q*) models the uncertainties in the state transition process, assuming Gaussian noise. It is a diagonal matrix with variances for each state variable. The values used for the evaluation of the airgun localization system in *Q* were the following:

$$Q = \begin{bmatrix} \sigma_x^2&0&0&0&0&0&0&0&0&0&0&0\\ 0&\sigma_y^2&0&0&0&0&0&0&0&0&0&0\\ 0&0&\sigma_{depth}^2&0&0&0&0&0&0&0&0&0\\ 0&0&0&\sigma_{roll}^2&0&0&0&0&0&0&0&0\\ 0&0&0&0&\sigma_{pitch}^2&0&0&0&0&0&0&0\\ 0&0&0&0&0&\sigma_{yaw}^2&0&0&0&0&0&0\\ 0&0&0&0&0&0&\sigma_{x/dt}^2&0&0&0&0&0\\ 0&0&0&0&0&0&0&\sigma_{y/dt}^2&0&0&0&0\\ 0&0&0&0&0&0&0&0&\sigma_{z/dt}^2&0&0&0\\ 0&0&0&0&0&0&0&0&0&\sigma_{roll/dt}^2&0&0\\ 0&0&0&0&0&0&0&0&0&0&\sigma_{pitch/dt}^2&0\\ 0&0&0&0&0&0&0&0&0&0&0&\sigma_{yaw/dt}^2 \end{bmatrix}$$

1 - Position variance σ_x^2 and σ_y^2 for the ahoi acoustic modem state variables it was considered its means absolute distance error in the range of 6cm to 16 cm (Reference [34, 35]):

$$\sigma_x = \sigma_y = 0.11\, m = \sigma_x^2 = 0.012\, m^2$$

2 – Orientation variance σ_{roll}^2, σ_{pitch}^2 and σ_{yaw}^2 for the IMU's orientation state variables roll, pitch and yaw considering the sensor specs manual:

$$\sigma_{roll} = \sigma_{pitch} = 0.2^{\circ} = 0.0035\, rad = \sigma_{roll}^2 = 0.000012\, rad^2$$

$$\sigma_{yaw} = 0.5^{\circ} = 0.0087\, rad = \sigma_{roll}^2 = 0.000075\, rad^2$$

3 – Linear velocity variance $\sigma_{x/dt}^2$, $\sigma_{y/dt}^2$ and $\sigma_{z/dt}^2$ for the IMU's linear velocity state variables *x/dt*, *y/dt*, *z/dt*:

$$\sigma_{x/dt} = \sigma_{y/dt} = \sigma_{z/dt} = 0.0125 \frac{m}{s} = \sigma_{x/dt}^2 = (0.00016\, \frac{m}{s})^2$$

4 – Angular acceleration variance $\sigma_{roll/dt}^2$, $\sigma_{pitch/dt}^2$ and $\sigma_{yaw/dt}^2$ for the IMU's angular acceleration state variables *roll/dt*, *pitch/dt* and *yaw/dt*:

$$\sigma_{roll/dt} = \sigma_{pitch/dt} = \sigma_{yaw/dt} = 2.908x10^{-6} \frac{rad}{s} = \sigma_{roll/dt}^2 = (8.46x10^{-12}\, \frac{rad}{s})^2$$

5 – Depth variance σ_{depth}^2 for the depth sensor state variable depth *z*:

$$\sigma_{depth} = 0.0025\, m = \sigma_{depth}^2 = 6.25x10^{-6}\, m^2$$

$$Q = \begin{bmatrix} 0.012 & 0 & 0 & 0 & 0 & 0 & 0 & 0 & 0 & 0 & 0 & 0 \\ 0 & 0.012 & 0 & 0 & 0 & 0 & 0 & 0 & 0 & 0 & 0 & 0 \\ 0 & 0 & 6.25x10^{-6} & 0 & 0 & 0 & 0 & 0 & 0 & 0 & 0 & 0 \\ 0 & 0 & 0 & 0.000012 & 0 & 0 & 0 & 0 & 0 & 0 & 0 & 0 \\ 0 & 0 & 0 & 0 & 0.00012 & 0 & 0 & 0 & 0 & 0 & 0 & 0 \\ 0 & 0 & 0 & 0 & 0 & 0.000075 & 0 & 0 & 0 & 0 & 0 & 0 \\ 0 & 0 & 0 & 0 & 0 & 0 & 0.00016 & 0 & 0 & 0 & 0 & 0 \\ 0 & 0 & 0 & 0 & 0 & 0 & 0 & 0.00016 & 0 & 0 & 0 & 0 \\ 0 & 0 & 0 & 0 & 0 & 0 & 0 & 0 & 0.00016 & 0 & 0 & 0 \\ 0 & 0 & 0 & 0 & 0 & 0 & 0 & 0 & 0 & 8.46x10^{-12} & 0 & 0 \\ 0 & 0 & 0 & 0 & 0 & 0 & 0 & 0 & 0 & 0 & 8.46x10^{-12} & 0 \\ 0 & 0 & 0 & 0 & 0 & 0 & 0 & 0 & 0 & 0 & 0 & 8.46x10^{-12} \end{bmatrix}$$

The *R* matrix represents the measurement noise covariance for each sensor, and the values for *R* depend on the characteristics of the sensors and their expected accuracy. The values used for the EKF measurement noise covariance used for the evaluation of the airgun localization system were the following:

$$R = \begin{bmatrix} \sigma_x^2 & 0 & 0 & 0 & 0 & 0 & 0 & 0 & 0 & 0 & 0 & 0 \\ 0 & \sigma_y^2 & 0 & 0 & 0 & 0 & 0 & 0 & 0 & 0 & 0 & 0 \\ 0 & 0 & \sigma_{depth}^2 & 0 & 0 & 0 & 0 & 0 & 0 & 0 & 0 & 0 \\ 0 & 0 & 0 & \sigma_{roll}^2 & 0 & 0 & 0 & 0 & 0 & 0 & 0 & 0 \\ 0 & 0 & 0 & 0 & \sigma_{pitch}^2 & 0 & 0 & 0 & 0 & 0 & 0 & 0 \\ 0 & 0 & 0 & 0 & 0 & \sigma_{yaw}^2 & 0 & 0 & 0 & 0 & 0 & 0 \\ 0 & 0 & 0 & 0 & 0 & 0 & \sigma_{x/dt}^2 & 0 & 0 & 0 & 0 & 0 \\ 0 & 0 & 0 & 0 & 0 & 0 & 0 & \sigma_{y/dt}^2 & 0 & 0 & 0 & 0 \\ 0 & 0 & 0 & 0 & 0 & 0 & 0 & 0 & \sigma_{z/dt}^2 & 0 & 0 & 0 \\ 0 & 0 & 0 & 0 & 0 & 0 & 0 & 0 & 0 & \sigma_{roll/dt}^2 & 0 & 0 \\ 0 & 0 & 0 & 0 & 0 & 0 & 0 & 0 & 0 & 0 & \sigma_{pitch/dt}^2 & 0 \\ 0 & 0 & 0 & 0 & 0 & 0 & 0 & 0 & 0 & 0 & 0 & \sigma_{yaw/dt}^2 \end{bmatrix}$$

Each diagonal term represents the noise variance σ^2 for a specific measurement, and the matrix components are as follows:

1 - Position variance σ_x^2 and σ_y^2 : Noise in the 2D measurements from the acoustic modem.

The positional accuracy of the ahoi acoustic modem is in the range of 60 cm to 75 cm (Reference [34])

$$\sigma_x = \sigma_y = 0.675\, m = \sigma_x^2 = 0.456\, m^2$$

2 – Orientation variance σ_{roll}^2, σ_{pitch}^2 and σ_{yaw}^2 : Noise in the IMU's orientation roll, pitch, and yaw measurements.

$$\sigma_{roll} = \sigma_{pitch} = 0.5^{\circ} = 0.008\, rad = \sigma_{roll}^2 = 0.000064\, rad^2$$

$$\sigma_{yaw} = 1.5^{\circ} = 0.026\, rad = \sigma_{roll}^2 = 0.00068\, rad^2$$

3 – Linear velocity variance $\sigma_{x/dt}^2$, $\sigma_{y/dt}^2$ and $\sigma_{z/dt}^2$: Noise in the IMU's linear velocity measurements

$$\sigma_{x/dt} = \sigma_{y/dt} = \sigma_{z/dt} = 0.05 \frac{m}{s} = \sigma_{x/dt}^2 = (0.0025 \frac{m}{s})^2$$

4 – Angular acceleration variance $\sigma_{roll/dt}^2$, $\sigma_{pitch/dt}^2$ and $\sigma_{yaw/dt}^2$: Noise in the IMU's angular acceleration measurements.

$$\sigma_{roll/dt} = \sigma_{pitch/dt} = \sigma_{yaw/dt} = 2.908x10^{-5} \frac{rad}{s} = \sigma_{roll/dt}^2 = (8.47x10^{-9} \frac{rad}{s})^2$$

5 – Depth variance σ_{depth}^2 : Noise in the depth sensor measurements

$$\sigma_{depth} = 0.02\, m = \sigma_{depth}^2 = 0.0004\, m^2$$

$$R = \begin{bmatrix} 0.456 & 0 & 0 & 0 & 0 & 0 & 0 & 0 & 0 & 0 & 0 & 0 \\ 0 & 0.456 & 0 & 0 & 0 & 0 & 0 & 0 & 0 & 0 & 0 & 0 \\ 0 & 0 & 0.0004 & 0 & 0 & 0 & 0 & 0 & 0 & 0 & 0 & 0 \\ 0 & 0 & 0 & 0.000064 & 0 & 0 & 0 & 0 & 0 & 0 & 0 & 0 \\ 0 & 0 & 0 & 0 & 0.000064 & 0 & 0 & 0 & 0 & 0 & 0 & 0 \\ 0 & 0 & 0 & 0 & 0 & 0.00068 & 0 & 0 & 0 & 0 & 0 & 0 \\ 0 & 0 & 0 & 0 & 0 & 0 & 0.0025 & 0 & 0 & 0 & 0 & 0 \\ 0 & 0 & 0 & 0 & 0 & 0 & 0 & 0.0025 & 0 & 0 & 0 & 0 \\ 0 & 0 & 0 & 0 & 0 & 0 & 0 & 0 & 0.0025 & 0 & 0 & 0 \\ 0 & 0 & 0 & 0 & 0 & 0 & 0 & 0 & 0 & 8.47x10^{-9} & 0 & 0 \\ 0 & 0 & 0 & 0 & 0 & 0 & 0 & 0 & 0 & 0 & 8.47x10^{-9} & 0 \\ 0 & 0 & 0 & 0 & 0 & 0 & 0 & 0 & 0 & 0 & 0 & 8.47x10^{-9} \end{bmatrix}$$

All off-diagonal terms are zero because measurements are assumed to be independent (uncorrelated).

In brief, the observation matrix H directly maps all measurements to their corresponding states (identity matrix), the process noise covariance matrix Q accounts for uncertainties in state evolution, and the observation noise covariance matrix R accounts for uncertainties in sensor measurements.

2 – Multilateration algorithm used for the airgun localization system

Multilateration is a positioning method that determines the location of a target (a mobile anchor) based on distance measurements to multiple reference points (fixed anchors). For the underwater testing of the airgun localization system, the components used for the multilateration method were the following:

- Four acoustic modems with known positions were used $(x_1, y_1), (x_2, y_2), (x_3, y_3), (x_4, y_4)$.
- An acoustic modem installed in the watertight enclosure (mobile anchor) (Fig. 4b in the manuscript) whose position (x, y) is to be determined.
- The system measures distances between the mobile and fixed anchors using the two-way time-of-flight ranging (TWR) method.

A single packet was used to collect distance from all fixed anchors. First, the mobile acoustic modem (mobile anchor) sends as a broadcast an empty-ranging request to the fixed anchors at time t_1 .Second, once the anchors receive the request, the anchors reply with a consecutive ranging response after a known delay Δt. Anchor A replied first, followed by anchors B, C and D with a single, double and triple delay (Δt= 750 ms) [34], respectively, to avoid interference between anchor ranging responses. The mobile anchor records the round-trip time t_{round} when it receives the reply from the fixed anchors.

The one-way travel time $t_{one-way}$ is calculated as follows:

$$t_{one-way} = \frac{t_{round} - \Delta t}{2} \quad (8)$$

Then the distance is calculated:

$$d = v \; x \; t_{one-way} \quad (9)$$

Where v is the speed of sound in water (v = 1500 m/s).

The localization using multilateration using the distances (d_1, d_2, d_3, d_4) to the four fixed anchors (Fig. 5d in the manuscript), the position of the mobile anchor (x_i, y_i) is calculated by solving a system of nonlinear equations. For each fixed anchor, the equation of a circle is:

$$(x - x_i)^2 + (y - y_i)^2 = d_i^2 \quad (10)$$

Where (x_i, y_i) is the position of each fixed anchor and d_i is the distance to each of the fixed anchors.

These equations represent the set of all possible locations of the mobile anchor relative to each fixed anchor. The mobile anchor's position is at the intersection of these circles:

$$(x - x_1)^2 + (y - y_1)^2 = d_1^2 \quad (11)$$

$$(x - x_2)^2 + (y - y_2)^2 = d_2^2 \quad (12)$$

$$(x - x_3)^2 + (y - y_3)^2 = d_3^2 \tag{13}$$

$$(x - x_4)^2 + (y - y_4)^2 = d_4^2 \tag{14}$$

To simplify solving the nonlinear equations, one can linearize them using algebraic manipulation. Subtract the equation for the first anchor from the others as follows:

$$(x - x_2)^2 + (y - y_2)^2 - (x - x_1)^2 + (y - y_1)^2 = d_2^2 - d_1^2 \tag{15}$$

Expanding and simplifying yields a linear equation. Repeating this for all the anchors results in a linear system, which can be solved using matrix methods. However, this system for the airgun localization algorithm described in this manuscript was solved numerically using nonlinear optimization through iterative solvers implemented in Python using scipy.optimize. The following image shows an example of a multilateration simulation using Scipy.optimize simulated done before we run the airgun localization system test in the different environments (air conditions and underwater conditions) described in the manuscript:

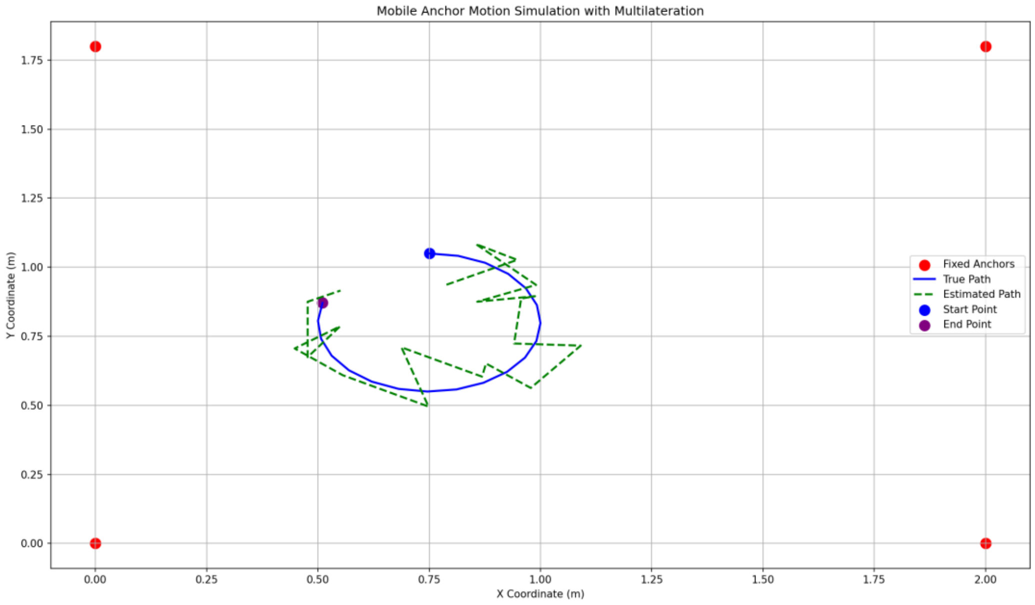

Fig. 1. Mobile anchor motion simulation with multilateration of a system using four fixed anchors and one mobile anchor in underwater conditions (simulated with small noise added to the estimated distances to mimic underwater environment inaccuracies).

3 - Localization error overtime of the airgun localization system testing in two different controlled environments (air conditioning using the UR3 robot tool and underwater conditions using the XY linear robot).

To calculate the localization error for each timestamp t, the Euclidean distance between the ground truth (UR3 robot or XY linear robot simulations) and estimated position (Airgun localization system position estimation) was calculated as follows:

$$e_t = \sqrt{(x_t^{true} - x_t^{est})^2 + (y_t^{true} - y_t^{est})^2} \tag{16}$$

Where $x_t^{true}$and y_t^{true} are the ground truth coordinates, x_t^{est} and y_t^{est} estimated coordinates, and e_t the localization error at time t. The calculation of the mean localization error of the system over time was done by computing the average of localization errors at all time points as follows:

$$\mu = \frac{1}{N} \sum_{i=1}^{N} e_t \tag{17}$$

Where N is the total number of time points. The standard deviation σ of the localization error over time quantifies the variability of errors was calculated using the following equation:

$$\sigma = \sqrt{\frac{1}{N} \sum_{i=1}^{N} (e_t - \mu)^2} \tag{18}$$

The following Figure 2 a-d illustrates the total localization error over time (X-Y) of the system tested in air and underwater conditions through different movement simulations from the depicted spatial plots. Figure 6 a-d in the manuscript illustrates the baseline trajectory position and estimated trajectory position based on the performance of the airgun localization system.

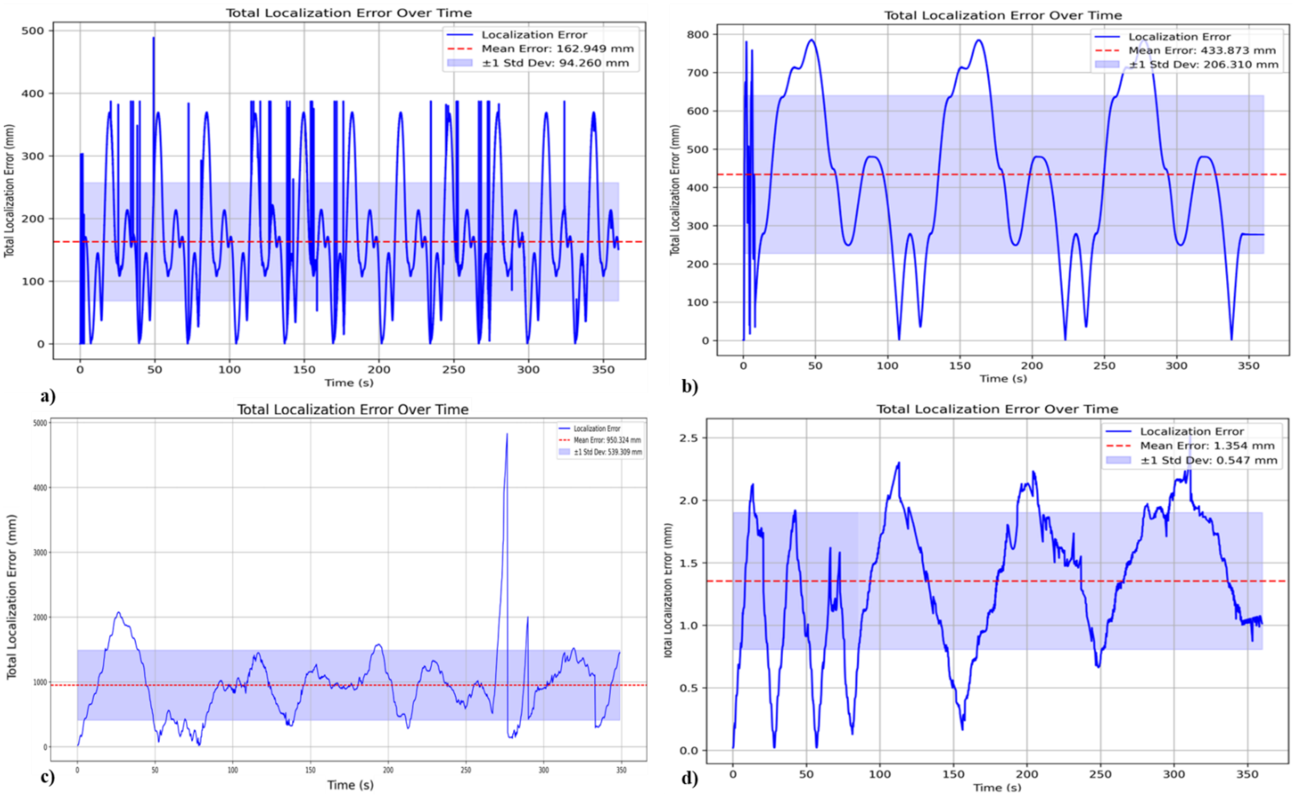

Fig. 2. a-b) Total localization error over time of the airgun localization system tested in a controlled environment through different simulations of movement using the UR3 robot tool. c-d) Total localization error over time of the airgun localization system tested in underwater conditions using the XY linear robot as baseline (see Appendix Video 1).

The independent error over time, separately for the X and Y axes, was calculated by the following equations:

$$e_t = |x_t^{true} - x_t^{est}| \quad (19)$$

$$e_t = |y_t^{true} - y_t^{est}| \quad (20)$$

These errors independently measure the deviation along each axis and can reveal axis-specific biases or inaccuracies. The mean error for the X and Y axes independently was calculated as follows:

$$\mu_x = \frac{1}{N} \sum_{i=1}^{N} |x_t^{true} - x_t^{est}| \quad (21)$$

$$\mu_y = \frac{1}{N} \sum_{i=1}^{N} |y_t^{true} - y_t^{est}| \quad (22)$$

The following Figure 3 a-d illustrates the localization error over time focused independently on the X and Y axes of the system tested in air and underwater conditions through different simulations of movement from the depicted spatial plots. Figure 6 a-d in the manuscript illustrates the baseline trajectory position and estimated trajectory position based on the performance of the airgun localization system.

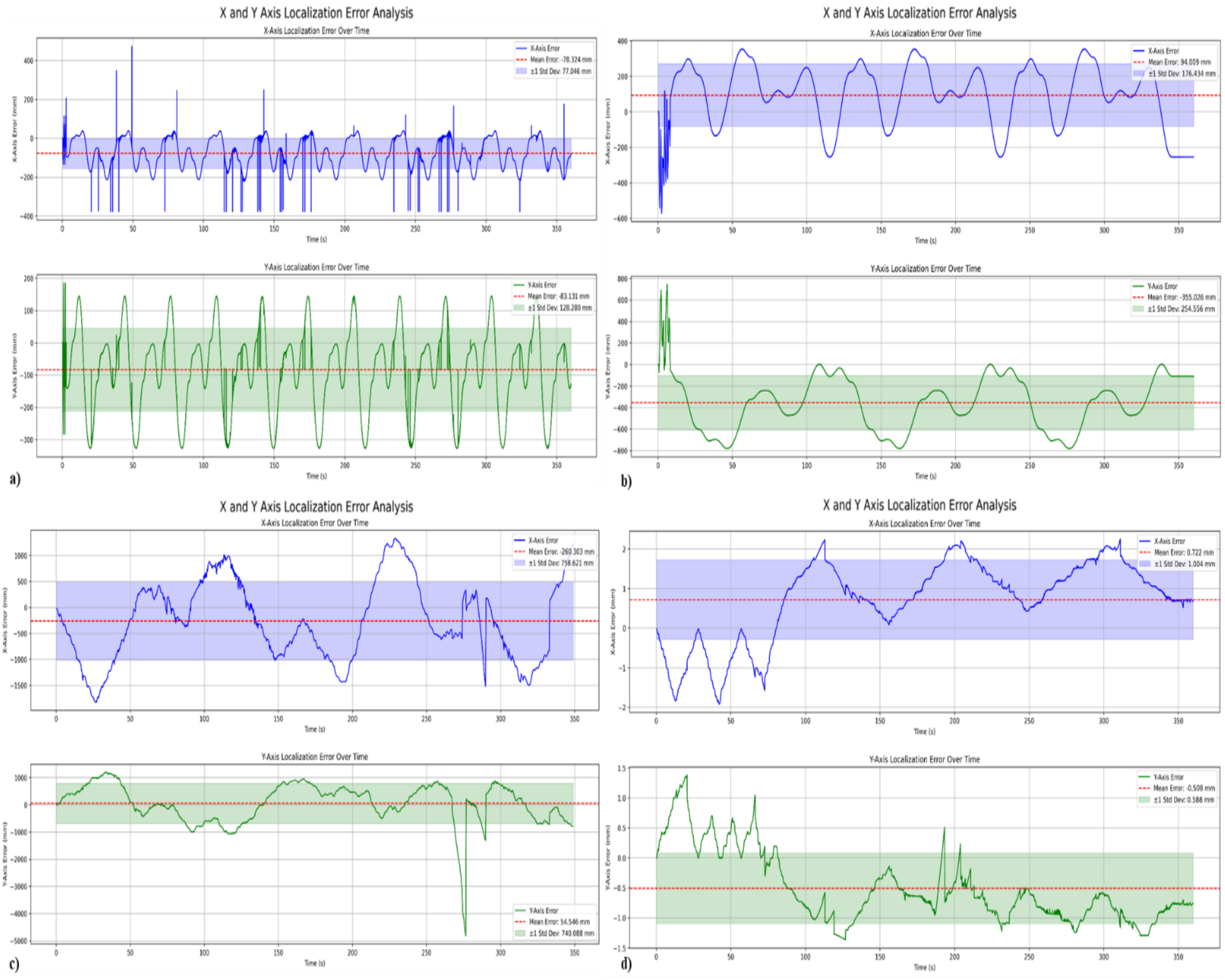

Fig. 3. a-b) Localization error over time of the airgun localization system in the X and Y axes, tested in a controlled environment through different simulations of movement using the UR3 robot tool. c-d) Total localization error over time of the airgun localization system in the X and Y axis, tested underwater using the XY linear robot as baseline (see Appendix Video 1).
